# Tyrosine phosphatase STEP is a key regulator of glutamate-induced prostaglandin E_2_ release from neurons

**DOI:** 10.1016/j.jbc.2021.100944

**Published:** 2021-07-08

**Authors:** Sathyanarayanan Rajagopal, Ranjana Poddar, Surojit Paul

**Affiliations:** Department of Neurology, University of New Mexico Health Sciences Center, University of New Mexico, Albuquerque, New Mexico, USA

**Keywords:** excitotoxicity, NMDA receptor, tyrosine phosphatase, STEP, p38 MAPK, neuroinflammation, cyclooxygenase-2, NF-κB, prostaglandin, COX-2, cyclooxygenase-2, cPLA2, cytosolic phospholipase A2, IκB, inhibitor of nuclear factor-κB, NF-κB, nuclear factor-κB, NMDA, N-methyl-D-aspartic acid, NMDARs, NMDA receptors, p38 MAPK, p38 mitogen-activated protein kinase, PGE_2_, prostaglandin E_2_, STEP, striatal-enriched phosphatase

## Abstract

The neuron-specific tyrosine phosphatase striatal-enriched phosphatase (STEP) is emerging as a key regulator of excitotoxicity, which is involved in the pathogenesis of both acute and chronic neurological diseases. However, the intracellular mechanisms that are regulated by STEP to confer neuroprotection against excitotoxic insults are not well understood. The present study investigates the role of STEP in regulating neuronal release of the proinflammatory prostanoid prostaglandin E_2_ (PGE_2_), which is associated with a wide range of pathological conditions. The findings show that glutamate-mediated activation of the N-methyl-D-aspartic acid receptor in STEP-deficient neurons leads to rapid and sustained increase in the phosphorylation of p38 mitogen-activated protein kinase (p38 MAPK), a signaling molecule involved in the production of inflammatory mediators. Such sustained p38 MAPK activation increases the activity of cytosolic phospholipase A_2_, which catalyzes the release of arachidonic acid, the initial substrate for PGE_2_ biosynthesis. Sustained p38 MAPK activation also induces nuclear factor-κB–mediated increase in expression of cyclooxygenase-2 that is involved in the conversion of arachidonic acid to prostanoids, resulting in enhanced biosynthesis and release of PGE_2_ from neurons. Restoration of STEP function with a STEP mimetic (TAT-STEP-myc peptide) significantly decreases the activation of p38 MAPK–mediated cytosolic phospholipase A_2_/cyclooxygenase-2/PGE_2_ signaling cascade. This study identifies an important mechanism involved in the neuronal release of the proinflammatory mediator PGE_2_ after excitotoxic insult and highlights for the first time the immunomodulatory ability of a neuronal tyrosine phosphatase.

Glutamate is the major excitatory neurotransmitter in the brain and is involved virtually in all activities of the central nervous system. Under physiological conditions, extracellular glutamate concentration in the brain is maintained in the low micromolar range by the excitatory amino acid transporters. However, an increase in extracellular glutamate concentration under pathological conditions leads to excessive activation of glutamate receptors in the nerve cells, resulting in excitotoxicity, which plays an important role in a range of neurological disorders (1, 2). These include acute neurological conditions such as ischemic stroke and traumatic brain injury as well as chronic neurodegenerative diseases such as Huntington’s disease and amyotrophic lateral sclerosis (1). Glutamate can bind to the ionotropic glutamate receptor subtypes N-methyl-D-aspartic acid (NMDA) and α-amino-3-hydroxy-5-methyl-4-isoxazolepropionic acid, kainate, and the metabotropic family of glutamate receptors (3, 4). However, the excitotoxic effects of glutamate are mediated primarily through the NMDA subtype of ionotropic glutamate receptors, which has the highest affinity for glutamate (5). Excessive stimulation of neuronal NMDA receptors (NMDARs) during an excitotoxic insult leads to intracellular Ca^2+^ overload and activation of a deleterious cascade of events resulting in neurotoxicity that eventually leads to brain damage. Although other ionotropic glutamate receptors and metabotropic glutamate receptors can modulate intracellular calcium ion in certain situations, they are less frequently associated with excitotoxicity (6).

It is evident from earlier studies that the stress-activated kinase, p38 mitogen-activated protein kinase (p38 MAPK), is a critical mediator of neuronal injury in both acute and chronic neurological disorders (7, 8, 9, 10, 11). Several studies have also shown that p38 MAPK plays a role in glutamate-mediated neuronal excitotoxicity (12, 13, 14). Another potentially important finding is that the brain-enriched and neuron-specific tyrosine phosphatase striatal-enriched phosphatase (STEP) is a key regulator of p38 MAPK phosphorylation and activation in neurons (13). STEP, also known as Ptpn5, is expressed specifically in neurons of the striatum, cortex, and hippocampus (15, 16). STEP_61_ is the membrane-bound isoform of the STEP family that is ubiquitously expressed in the brain and is the predominant isoform expressed in primary cultured neurons (16, 17, 18). It is a signaling molecule downstream of NMDAR stimulation, whose activation after an excitotoxic insult provides initial neuroprotection by dephosphorylating and downregulating p38 MAPK activity, whereas degradation of active STEP over time leads to secondary activation of p38 MAPK, resulting in the progression of neuronal injury and brain damage (11, 13). In addition, it has also been reported that the activity of STEP in the brain decreases with aging (19), suggesting that the loss of this neuroprotective signal could be a contributing factor for the increased vulnerability of the elderly population to excitotoxicity associated neurological diseases (20, 21). Consistent with this interpretation, studies in animal models of cerebral stroke have shown that that loss of endogenous STEP leads to exacerbation of ischemic brain damage (11, 22). However, the intracellular signaling cascades that are involved in accelerating the progression of excitotoxic neural injury in the absence of STEP are not clearly understood. The goal of the present study is to evaluate the outcome of excitatory NMDAR stimulation in neuronal cultures obtained from WT and STEP KO mice. Our findings show that an excitatory insult in the absence of STEP triggers an inflammatory response through increased neuronal release of the proinflammatory prostanoid, prostaglandin E_2_ (PGE_2_), which involves p38 MAPK–mediated increased cytosolic phospholipase A_2_ (cPLA2) activation and nuclear factor-κB (NF-κB)-dependent cyclooxygenase-2 (COX-2) expression. The findings present the novel concept that the tyrosine phosphatase STEP promotes neuroprotection against excitotoxic insult through regulation of neuroinflammatory responses.

## Results

### Stimulation of neurons with glutamate in the absence of STEP leads to sustained p38 MAPK phosphorylation

**Figure 1:**
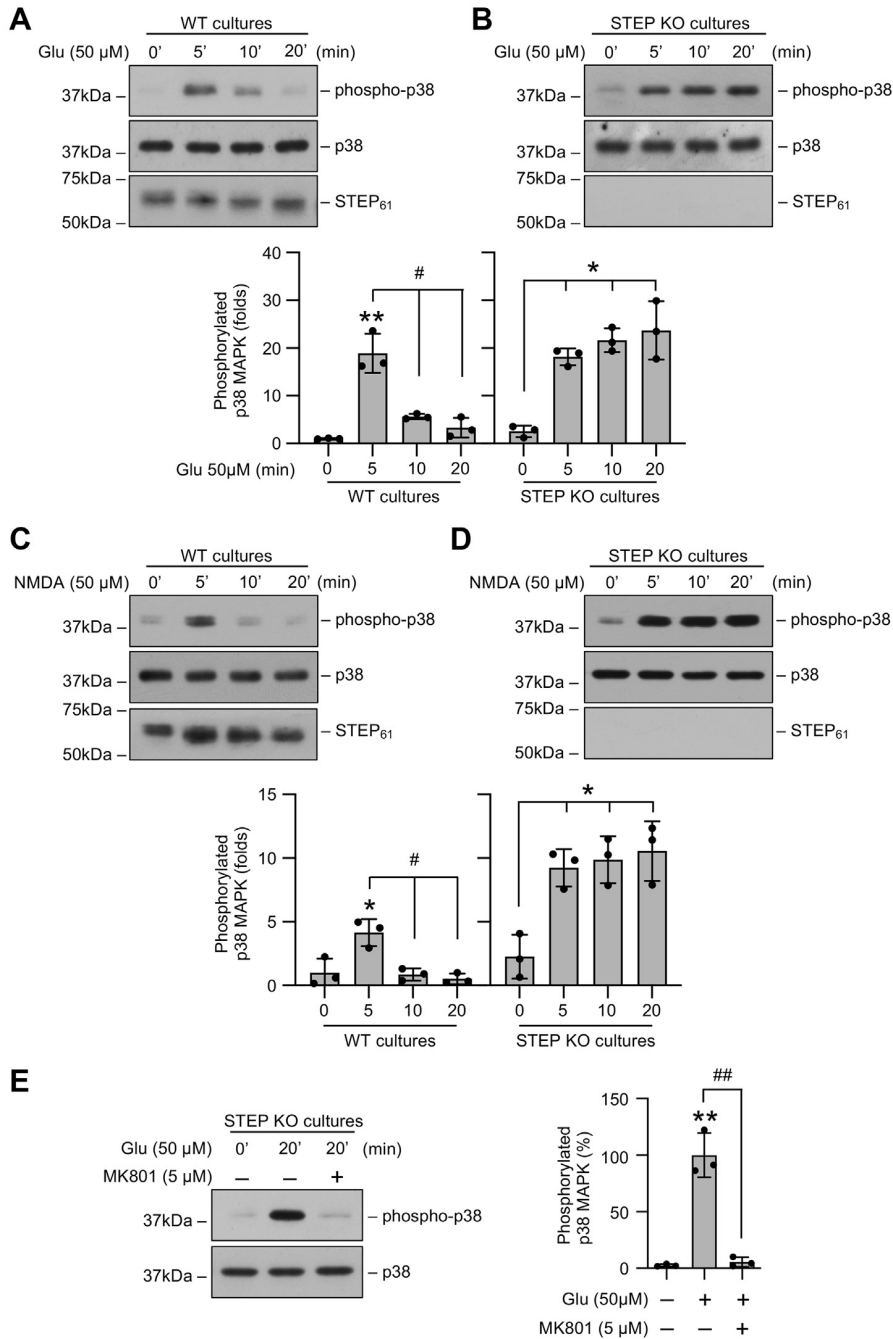
**Glutamate induces sustained p38 MAPK phosphorylation in STEP-deficient neurons.** Neuronal cultures from (*A* and *C*) WT and (*B* and *D*) and STEP KO mice were treated with 50 μM glutamate (Glu) or 50 μM NMDA for the specified times. *A*–*D*, equal amounts of protein from each sample were analyzed by immunoblot analysis using anti-phospho-p38 (*top*), anti-p38 (*middle*), and anti-STEP (*bottom*) antibodies. *E*, neuronal cultures from STEP KO mice were treated with glutamate (50 μM) for 20 min in the absence or presence of MK801 (5 μM). Protein extracts were analyzed by immunoblotting with anti-phospho-p38 (*top*) and anti-p38 (*bottom*) antibodies. Corresponding bar diagrams represent quantitative analysis of p38 MAPK phosphorylation as the mean ± SD (n = 3). ∗*p* < 0.01 and ∗∗*p* < 0.001 compared with the untreated control. Statistical analysis has been performed using ANOVA with Tukey’s post hoc test. ^#^*p* < 0.001 and ^##^*p* < 0.0001 from 5 min glutamate treatment. NMDA, N-methyl-D-aspartic acid; p38 MAPK, p38 mitogen-activated protein kinase; STEP, striatal-enriched phosphatase.

**Figure 2:**
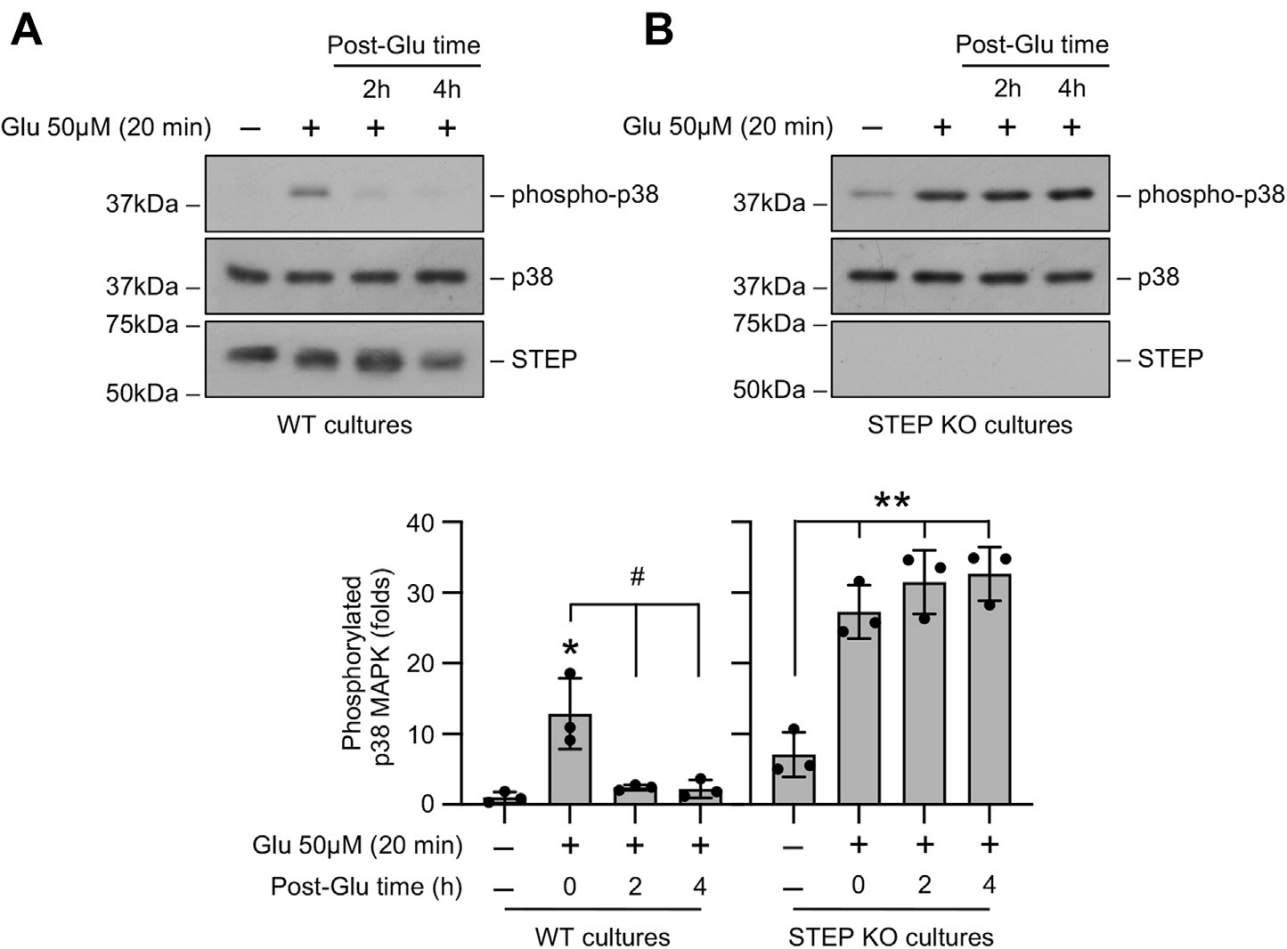
**Sustained p38 MAPK phosphorylation in STEP-deficient neurons in the postglutamate recovery phase.** Neuronal cultures from (*A*) WT and (*B*) and STEP KO mice were treated with 50 μM glutamate (Glu) for 20 min and then maintained in their original medium for the specified times (post-Glu time). Protein extracts were analyzed by immunoblotting with anti-phospho-p38 (*top*), anti-p38 (*middle*), and anti-STEP (*bottom*) antibodies. Corresponding bar diagrams represent quantitative analysis of p38 MAPK phosphorylation as the mean ± SD (n = 3). Statistical analysis has been performed using ANOVA with Tukey’s post hoc test. ∗*p* < 0.01 and ∗∗*p* < 0.001 compared with the untreated control. ^#^*p* < 0.01 from 20 min glutamate treatment. p38 MAPK, p38 mitogen-activated protein kinase; STEP, striatal-enriched phosphatase.

In initial studies, corticostriatal neuronal cultures (12–14 days *in vitro*) from WT and STEP KO mice were exposed to an excitotoxic insult to examine the consequences of STEP gene deletion on p38 MAPK phosphorylation. For these experiments, neuronal cultures were treated briefly with 50 μM glutamate for 5, 10, or 20 min to assess acute effects or for 20 min followed by recovery (2 h and 4 h) to assess delayed effects. Immunoblot analysis of cell lysates from WT mice showed a rapid increase in p38 MAPK phosphorylation within 5 min of glutamate treatment that decreased to near basal levels by 20 min of the insult (Fig. 1*A*). The samples were then analyzed with an anti-STEP antibody to evaluate the activation of STEP_61_ through dephosphorylation, as detected by the downward shift in mobility of the STEP band (11, 13, 23). The findings show that glutamate treatment resulted in dephosphorylation and subsequent activation of STEP_61_ by 20 min (Fig. 1*A*). On the other hand, in the neurons obtained from STEP KO mice, p38 MAPK phosphorylation remained sustained throughout the duration of the insult, and as expected, no STEP protein was detectable (Fig. 1*B*). Treatment with NMDA (50 μM) resulted in a similar transient activation of p38 MAPK in neurons from WT mice and sustained activation of p38 MAPK in neurons from STEP KO mice (Fig. 1, *C* and *D*). To further confirm the contribution of the NMDAR in the sustained p38 MAPK phosphorylation observed in STEP KO mice neurons, in some experiments, neurons from STEP KO mice were treated with glutamate (50 μM, 20 min) in the presence of the selective NMDAR inhibitor, MK801 (5 μM). The findings showed that the increase in p38 MAPK phosphorylation observed 20 min after glutamate treatment was significantly reduced in the presence of MK801 (Fig. 1*E*). In subsequent studies, a comparative analysis of p38 MAPK phosphorylation during recovery (2 h and 4 h) after glutamate treatment (50 μM, 20 min) showed that in the neurons from WT mice, p38 MAPK phosphorylation remained at basal levels, whereas in the neurons from STEP KO mice, p38 MAPK phosphorylation remained elevated throughout the time period of the study (Fig. 2, *A* and *B*). STEP_61_ also remained dephosphorylated in WT mice neurons during recovery (Fig. 2*A*). Together these findings indicate that after an excitotoxic insult, activation of STEP_61_ in WT mice neurons limits the duration of p38 MAPK phosphorylation, while loss of endogenous STEP in STEP KO mice neurons leads to prolonged p38 MAPK phosphorylation.

### Sustained p38 MAPK phosphorylation in the absence of STEP enhances cPLA2 activation and COX-2 expression resulting in increased neuronal PGE_2_ release

**Figure 3:**
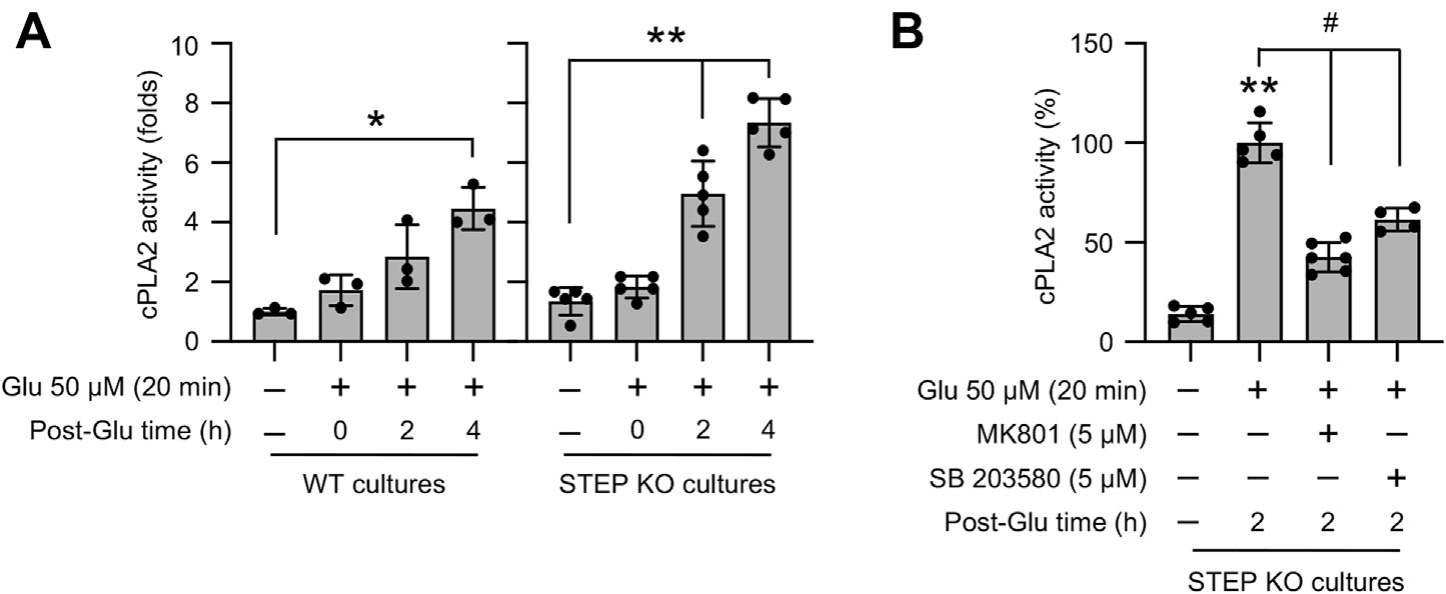
**Role of the NMDAR and p38 MAPK in glutamate-induced cPLA2 activation in STEP-deficient neurons.***A*, neuronal cultures from WT and STEP KO mice were treated with 50 μM glutamate (Glu) for 20 min and then maintained in their original medium for the specified times (post-Glu time). *B*, neuronal cultures from STEP KO mice were treated with 50 μM glutamate (Glu) for 20 min followed by recovery (post-Glu time) in the absence and presence of MK801 (5 μM) or SB 203580 (5 μM). *A* and *B*, equal amounts of protein from each sample were analyzed for cPLA2 activity using the enzymatic assay. Statistical analysis has been performed using ANOVA with Tukey’s post hoc test. Values are expressed as the mean ± SD (n = 3–6). ∗*p* < 0.001 and ∗∗*p* < 0.001 compared with the untreated control and ^#^*p* < 0.0001 from 2 h postglutamate time. cPLA2, cytosolic phospholipase A_2_; p38 MAPK, p38 mitogen-activated protein kinase; STEP, striatal-enriched phosphatase.

**Figure 4:**
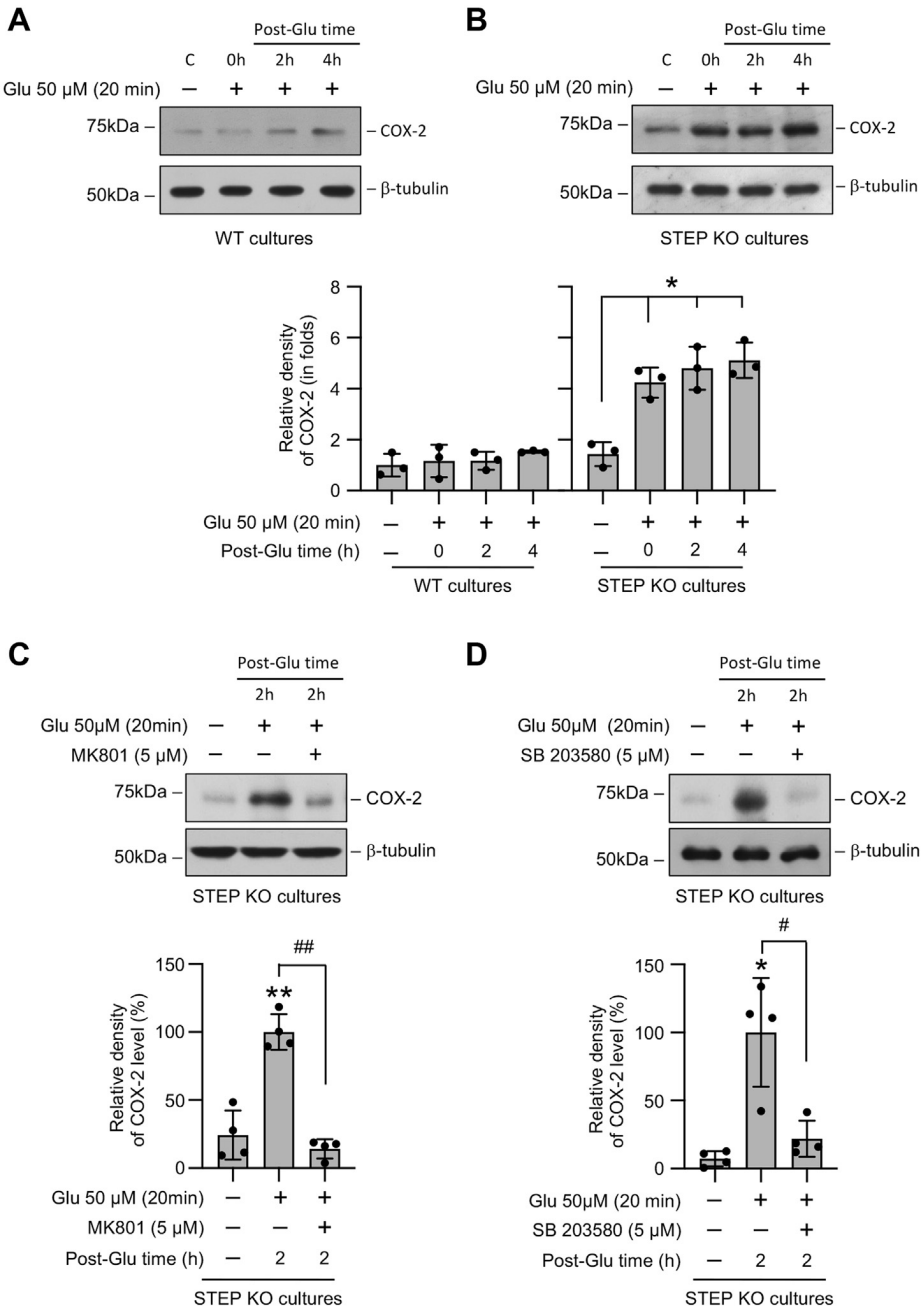
**Role of the NMDAR and p38 MAPK in glutamate-induced increase in the COX-2 protein level in STEP-deficient neurons.** Neuronal cultures from (*A*) WT and (*B*) STEP KO mice were treated with 50 μM glutamate (Glu) for 20 min and then maintained in their original medium for the specified times (post-Glu time). *C* and *D*, neuronal cultures from STEP KO mice were treated with 50 μM glutamate (Glu) for 20 min followed by recovery (post-Glu time) in the absence and presence of (*C*) MK801 (5 μM) or (*D*) SB 203580 (5 μM). Equal amounts of protein from each sample were analyzed by immunoblotting using anti-COX-2 (*top*) and anti-β-tubulin (*bottom*) antibodies. Corresponding bar diagrams represents quantitative analysis of COX-2 protein level as the mean ± SD (n = 3–4). Statistical analysis has been performed using ANOVA with Tukey’s post hoc test. ∗*p* < 0.001 and ∗∗*p* < 0.0001 compared with untreated control and ^#^*p* < 0.01 and ^##^*p* < 0.001 from 2 h postglutamate time. COX-2, cyclooxygenase-2; p38 MAPK, p38 mitogen-activated protein kinase; STEP, striatal-enriched phosphatase.

Earlier studies have indicated that excitotoxic stimulation of the ionotropic glutamate receptor in neurons could activate cPLA2, which catalyzes membrane phospholipids to release arachidonic acid, the initial substrate for PGE_2_ biosynthesis (24, 25, 26, 27). To evaluate the effect of an excitotoxic insult on cPLA2 activity, neuron cultures from WT and STEP KO mice were treated with glutamate (50 μM, 20 min) followed by recovery for varying time period (2 h and 4 h). Figure 3*A* shows a significant increase in cPLA2 activity in neurons from WT mice after 4 h of recovery. However, in neurons from STEP KO mice, the increase in cPLA2 activity was observed within 2 h of recovery, which remained elevated for the time period of the study. These findings suggest that deletion of STEP gene accelerates glutamate-mediated cPLA2 activation in neurons. To determine the role of the NMDAR and p38 MAPK in accelerating cPLA2 activity in the absence of STEP, the NMDAR inhibitor MK801 (5 μM) or the p38 MAPK inhibitor SB 203580 (5 μM) was added to neurons from STEP KO mice during glutamate treatment (50 μM, 20 min) and recovery (2 h). Figure 3*B* shows that application of either MK801 or SB 203580 significantly reduced cPLA2 activity observed 2 h after the insult. In a parallel series of experiments, we further evaluated the expression of COX-2, which is involved in the conversion of arachidonic acid to prostanoids. Figure 4*A* shows that the COX-2 level in neurons from WT mice remained unchanged during the insult and recovery, when compared with untreated control. However, in the neurons from the STEP KO mice, COX-2 level increased significantly within 20 min of the insult and remained elevated during the recovery phase (Fig. 4*B*). Pharmacological studies in neurons from STEP KO mice further showed that inhibition of the NMDAR with MK801 or p38 MAPK activation with SB 203580 attenuated glutamate-induced increase in the COX-2 level (Fig. 4, *C* and *D*).

**Figure 5:**
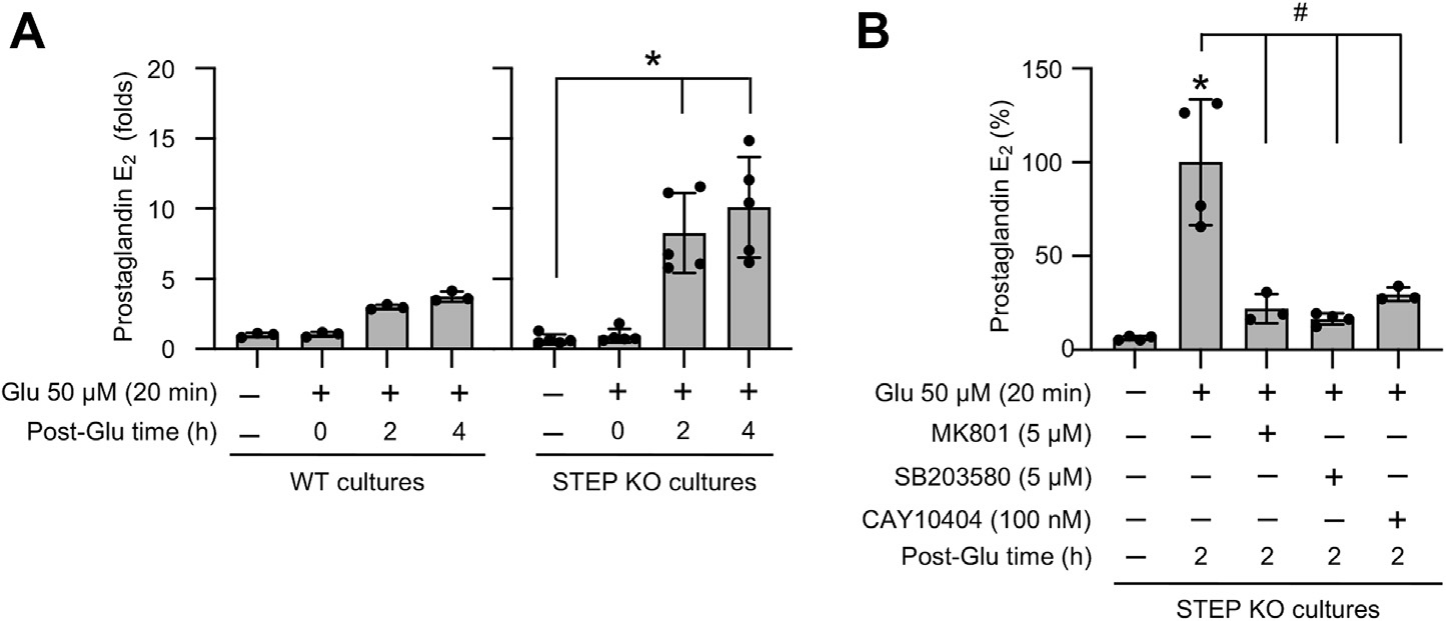
**Role of the NMDAR, p38 MAPK, and COX-2 in glutamate-induced increased PGE**_**2**_**release from STEP-deficient neurons.***A*, neuronal cultures from WT and STEP KO mice were treated with 50 μM glutamate (Glu) for 20 min and then maintained in their original medium for the specified times (post-Glu time). *B*, neuronal cultures from STEP KO mice were treated with 50 μM glutamate (Glu) for 20 min followed by recovery (post-Glu time) in the absence and presence of MK801 (5 μM), SB 203580 (5 μM), or CAY10404 (100 nM). *A* and *B*, equal amounts of culture media from each sample were analyzed for PGE_2_ level using ELISA. Statistical analysis has been performed using ANOVA with Tukey’s post hoc test. Values are expressed as the mean ± SD (n = 3–5). ∗*p* < 0.0001 compared with the untreated control and ^#^*p* < 0.001 from 2 h postglutamate time. STEP, striatal-enriched phosphatase.

To evaluate the effect of STEP gene deletion on glutamate-mediated PGE_2_ release in subsequent studies, neurons from WT and STEP KO mice were treated with glutamate (50 μM, 20 min), and the culture media from the neurons were analyzed for PGE_2_ level at different time points during recovery (2 h and 4 h). The results showed that glutamate treatment had no significant effect on PGE_2_ release from WT mice neurons. However, PGE_2_ release increased significantly from STEP KO mice neurons within 2 h after the insult, which remained elevated at 4 h after the insult (Fig. 5*A*). Additional studies investigated the effect of the NMDAR (MK801), p38 MAPK (SB 203580), and COX-2 (CAY10404) inhibitors on the release of PGE_2_ from STEP KO mice neurons. The findings demonstrated that glutamate-induced PGE_2_ release from STEP KO mice neurons was significantly reduced after inhibition of the NMDAR, p38 MAPK, or COX-2 (Fig. 5*B*), indicating the involvement of p38 MAPK/COX-2 signaling pathway in glutamate-NMDAR–induced neuronal PGE_2_ release in the absence of STEP.

### Glutamate-induced COX-2 expression and PGE_2_ release in the absence of STEP is regulated by NF-κB

**Figure 6:**
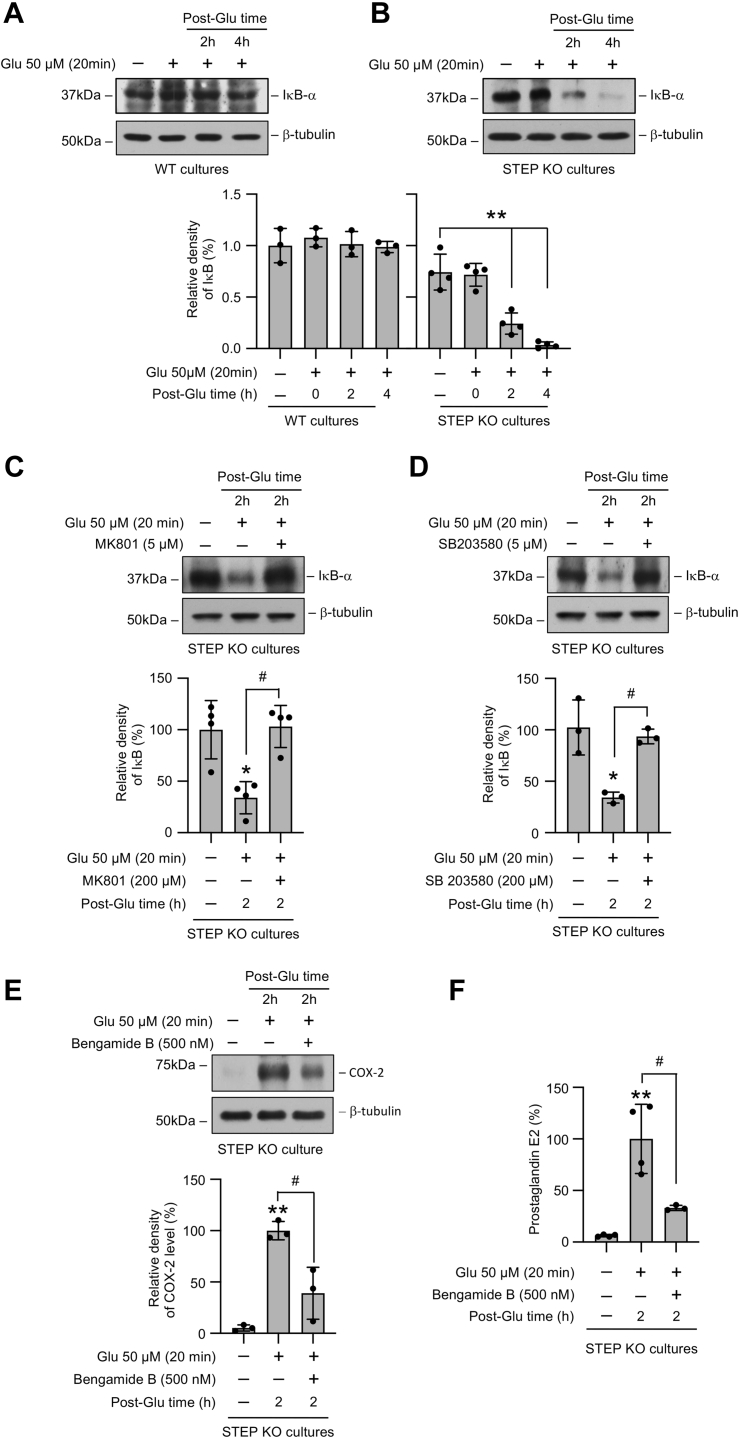
**Role of NF-κB in glutamate-induced increase in the COX-2 protein level in STEP-deficient neurons.***A* and *B*, neuronal cultures from (*A*) WT and (*B*) STEP KO mice were treated with 50 μM glutamate (Glu) for 20 min and then maintained in their original medium for the specified times (post-Glu time). *C*–*F*, neuronal cultures from STEP KO mice were treated with 50 μM glutamate (Glu) for 20 min followed by recovery (post-Glu time) in the absence and presence of (*C*) MK801 (5 μM), (*D*) SB 203580 (5 μM), or (*E* and *F*) Bengamide B (500 nM). Equal amounts of protein from each sample were analyzed by immunoblotting using (*A*–*D*) anti-IκBα (*top*) and anti-β-tubulin (*bottom*) antibodies, or (*E*) anti-COX-2 (*top*) and anti-β-tubulin (*bottom*) antibodies. Corresponding bar diagrams represents quantitative analysis of (*A*–*D*) IκBα or (*E*) COX-2 protein levels as the mean ± SD. *F*, equal amounts of culture media from each sample were analyzed for PGE_2_ level using ELISA. Statistical analysis has been performed using ANOVA with Tukey’s post hoc test. Values are expressed as the mean ± SD (n = 3–4). ∗*p* < 0.01 and ∗∗*p* < 0.001 compared with the untreated control and ^#^*p* < 0.01 from 2 h postglutamate time. COX-2, cyclooxygenase-2; IκB, inhibitor of nuclear factor-κB; STEP, striatal-enriched phosphatase.

Earlier studies have shown that the transcription factor NF-κB is a key regulator of COX-2 expression in different cell types (28, 29, 30, 31, 32), and the ionotropic glutamate receptor is one of the activators of NF-κB in neurons (33). To clarify the role of NF-κB in glutamate-NMDAR–induced activation of the COX-2/PGE_2_ signaling pathway in STEP KO mice neurons, we next evaluated the effect of glutamate on inhibitor of nuclear factor-κB (IκB) degradation, a seminal step in NF-κB activation (34, 35). Neuron cultures from WT and STEP KO mice were treated with 50 μM glutamate for 20 min followed by recovery (2 h and 4 h). Immunoblot analysis of neuronal lysates showed that brief exposure to glutamate had no effect on IκB level in the neurons obtained from WT mice, when compared with untreated control (Fig. 6*A*). However, in the neurons obtained from STEP KO mice, a significant decrease in IκB level was observed at 2 h and 4 h after the 20 min of insult (Fig. 6, *A* and *B*), indicating increased IκB degradation and NF-κB activation. Exposure to glutamate in the presence of inhibitors of the NMDAR or p38 MAPK effectively blocked glutamate-induced IκB degradation (Fig. 6, *C* and *D*). In additional studies, neurons were treated with glutamate in the presence of Bengamide B (500 nM), a potent inhibitor of NF-κB activation (35). Immunoblot analysis showed that coincubation with Bengamide B attenuated glutamate-induced increase in COX-2 protein level (Fig. 6*E*). Evaluation of PGE_2_ release in the culture medium obtained from the same experiments showed significant reduction in PGE_2_ release in the presence of Bengamide (Fig. 6*F*).

### Restoration of STEP signaling in STEP KO mice neurons attenuates glutamate-induced p38 MAPK activation, COX-2 expression, and PGE_2_ release

**Figure 7:**
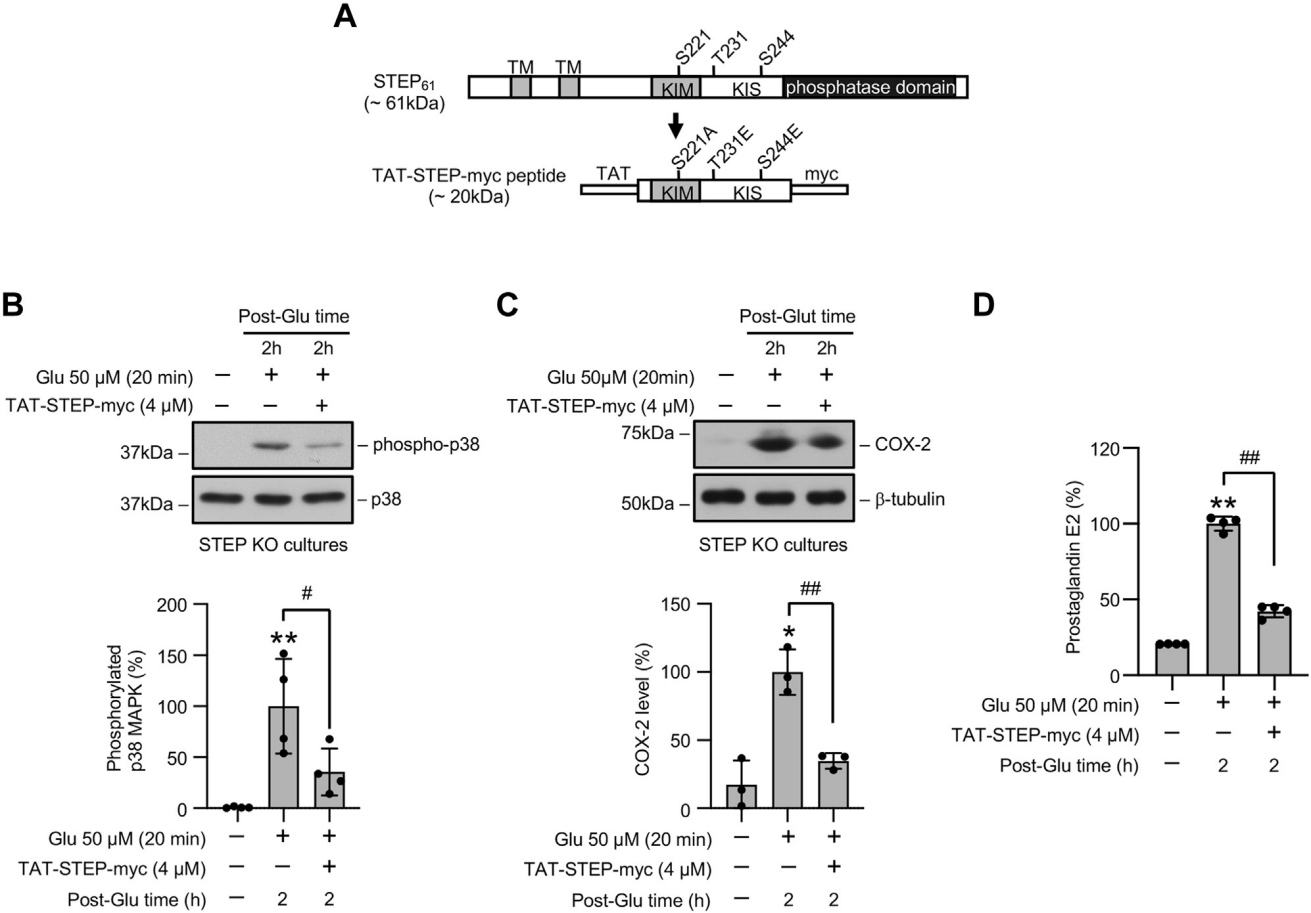
**A STEP mimetic attenuates glutamate-induced increase in p38 MAPK phosphorylation, COX-2 expression, and PGE**_**2**_**release in STEP-deficient neurons.***A*, schematic representation of TAT-STEP-Myc peptide generated from STEP_61_. The diagram of STEP_61_ shows the positions of the phosphatase domain, transmembrane domain (TM), kinase-interaction motif (KIM), kinase specificity sequence (KIS), and the phosphorylation sites in the KIM and KIS domains. The diagram of the TAT-STEP-Myc peptide (STEP mimetic), derived from STEP_61_, shows the positions of the TAT domain at the N terminus, myc-tag at the C terminus, the serine residue in the KIM domain that was mutated to alanine to allow the peptide to bind to its substrates, and the threonine and serine residues in the KIS domain, which were mutated to glutamic acid to render the peptide resistant to degradation. *B*–*D*, neuronal cultures from STEP KO mice were treated with 50 μM glutamate (Glu) for 20 min in the absence and presence of TAT-STEP-myc peptide and then maintained in their original medium for 2 h (post-Glu time). *B* and *C*, equal amounts of protein from each sample were analyzed by immunoblotting using anti-phospho-p38 (*top*) and anti-p38 (*bottom*) antibodies. Corresponding bar diagrams represent quantitative analysis of p38 MAPK phosphorylation as the mean ± SD (n = 3–4). *D*, equal amounts of culture media from each sample were analyzed for the PGE_2_ level using ELISA. Statistical analysis has been performed using ANOVA with Tukey’s post hoc test. Values are expressed as the mean ± SD (n = 4). ∗*p* < 0.01 and ∗∗*p* < 0.001 compared with the untreated control. ^#^*p* < 0.05 and ^##^*p* < 0.01 from 2 h post-glutamate time. COX-2, cyclooxygenase-2; p38 MAPK, p38 mitogen-activated protein kinase; STEP, striatal-enriched phosphatase.

To directly test the hypothesis that exposure to glutamate in the absence of endogenous STEP leads to upregulation of p38 MAPK/COX-2 signaling pathway resulting in increased PGE_2_ release, we generated a cell-permeable STEP mimetic (Fig. 7*A*, TAT-STEP-myc peptide) that constitutively binds to and inhibits p38 MAPK activation in neurons (11, 13). Neuron cultures from STEP KO mice were preincubated with the STEP-mimetic (4 μM) followed by a brief exposure to glutamate (20 min) and recovery (2 h). Immunoblot analysis showed that the application of the STEP-mimetic blocked the phosphorylation of p38 MAPK assessed at 2 h after recovery (Fig. 7*B*). COX-2 expression was also significantly reduced after peptide treatment (Fig. 7*C*). Evaluation of PGE_2_ release in the medium from the same experiments showed significant reduction in PGE_2_ release (Fig. 7*D*). These findings confirm the role of STEP in regulating a proinflammatory response in neurons after an excitotoxic insult.

## Discussion

**Figure 8:**
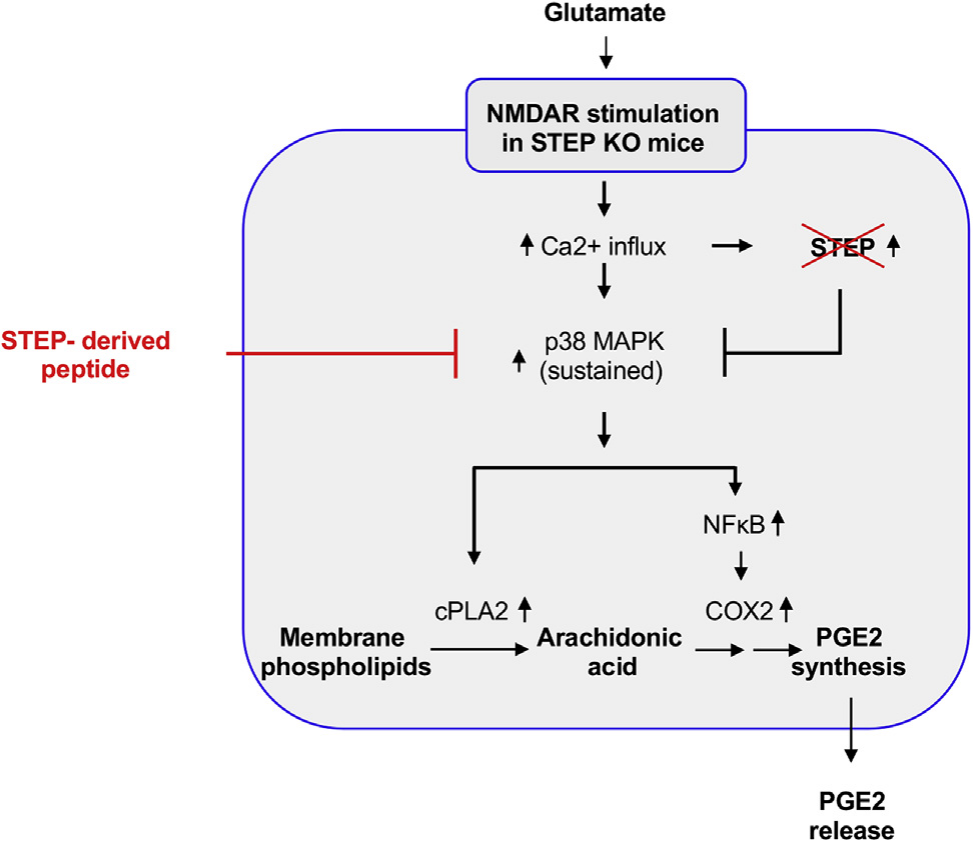
**Schematic representation of the signaling cascade regulated by STEP in glutamate-induced neuronal PGE**_**2**_**release.** COX-2, cyclooxygenase-2; cPLA2, cytosolic phospholipase A_2_; NF-κB, nuclear factor-κB; NMDAR, NMDA receptor; p38 MAPK, p38 mitogen-activated protein kinase; PGE_2_, prostaglandin E_2_; STEP, striatal-enriched phosphatase.

Previous studies have implicated a role of excitotoxicity in a variety of neuropathological conditions associated with aging, indicating that excitotoxicity could be a common pathogenic pathway in neurodegenerative disorders with distinctly different genetic etiology. The deleterious effects of excitotoxicity include impairment of intracellular Ca^2+^ buffering, generation of free radicals, and mitochondrial dysfunction. Emerging studies indicate that excitotoxicity can also trigger inflammatory response in the brain. However, the underlying mechanisms through which excessive activation of glutamate receptors could enhance inflammatory response in the brain is not well understood. The present study highlights the role of STEP as a key regulator of inflammatory response in neurons, after an excitotoxic insult. The findings show that in the absence of endogenous STEP, sustained p38 MAPK activation after an excitotoxic insult leads to increased cPLA2 activation and NF-κB-mediated COX-2 expression, resulting in biosynthesis and release of the proinflammatory mediator PGE_2_ from neurons, whereas restoration of STEP signaling with a STEP-derived peptide mimetic attenuates p38 MAPK/cPLA2/COX-2 mediated PGE_2_ release. A schematic representation of this signaling cascade is presented in Figure 8.

The characterization of the role of STEP in regulating the neuronal production of PGE_2_ reveals that STEP constitutes an important control point, restricting the early onset of inflammatory response after an insult. The distinctly different pattern of p38 MAPK signaling in neurons from WT and STEP KO mice could be attributed to the loss of STEP, as p38 MAPK is a substrate of STEP. In the absence of this inhibitory signal, sustained activation of p38 MAPK is the initial trigger in glutamate-NMDAR–mediated neuronal PGE_2_ synthesis and release. An early onset and substantial increase in the activity of cPLA2, the first enzyme involved in the synthesis of PGE_2_, was observed in neurons from STEP KO mice, and to a much lesser extent in neurons from the WT mice. However, NF-κB–mediated increase in the expression COX-2, the second enzyme involved in PGE_2_ biosynthesis, was observed only in STEP KO mice neurons. The activation of cPLA2 and the expression of COX-2 in different cell types are regulated by multiple signaling pathways, including p38 MAPK pathway, which has been found to be activated in several neurological disorders that includes ischemic stroke, Alzheimer’s disease, Parkinson’s disease, and amyotrophic lateral sclerosis (10, 11, 36, 37). Earlier studies have reported that p38 MAPK phosphorylates cPLA2 at Ser505 to augment its activity (38, 39). More recent studies showed that p38 MAPK activation enhances the stability of COX-2 mRNA, resulting in increased protein levels (40, 41). Consistent with these observations, we observed that inhibition of p38 MAPK activation attenuated both cPLA2 activation and COX-2 expression in STEP KO mice neurons. In neurons from STEP KO mice, we also observed sustained and significant increase in PGE_2_ release, which was attenuated by pharmacological inhibition of p38 MAPK, NF-κB, and COX-2. Taken together, these findings indicate that the consequences of p38 MAPK activation depend largely on the duration of its activation. A transient activation of p38 MAPK in neurons from WT mice fails to exert substantial effect on PGE_2_ synthesis. In contrast, the prolonged activation of p38 MAPK observed in neurons from STEP KO mice is crucial for the synergistic activation of cPLA2 and elevated expression of COX-2, which leads to robust increase in PGE_2_ synthesis.

The sustained phosphorylation of p38 MAPK in the absence of STEP and the attenuation of p38 MAPK phosphorylation after restoration of STEP signaling with a cell-permeable STEP mimetic reveal a novel mechanism of regulation of inflammatory response in neurons after an excitotoxic insult. In earlier studies, we have established that the interaction between STEP and its substrates is regulated by the phosphorylation of a critical serine residue within the kinase-interaction motif (KIM) domain of STEP. Dopamine/D1 receptor–mediated phosphorylation of this serine residue renders STEP inactive in terms of its ability to bind to its substrate (42). Dephosphorylation of this residue after glutamate/NMDAR stimulation allows STEP to bind to it substrates and inhibit their activity (13, 23). The specificity of the dephosphorylated form of STEP to bind to its substrates has been demonstrated in several earlier studies, where a critical serine residue (ser 221) in the KIM domain was either mutated to alanine to mimic the dephosphorylated form or to glutamic acid to mimic the phosphorylated form of STEP (13, 23, 43, 44, 45, 46). These studies demonstrated that mutation of the serine residue in the KIM domain to alanine allows STEP to bind constitutively to its substrates. In contrast, the mutant form of STEP where the serine residue in the KIM domain was converted to glutamic acid fails to bind to its substrates. Furthermore, WT STEP, which is basally phosphorylated at the serine residue in the KIM domain also fails to bind to its substrates. Based on these findings, a STEP-derived peptide (TAT-STEP-myc) was generated, where the serine residue in the KIM domain was mutated to allow the peptide to bind constitutively with its substrates, which includes p38 MAPK (13, 22). In addition, a threonine and a serine residue in the adjacent kinase specificity sequence were mutated to glutamic acid to maintain the stability of the peptide (43). In the present study, restoration of STEP signaling in STEP KO mice neurons with the application of this STEP-mimetic not only reduced p38 MAPK phosphorylation but also attenuated COX-2 expression and PGE_2_ synthesis. Excessive and persistent release of PGE_2_ in the brain has been associated with microglial activation and peripheral immune cell infiltration, the two cardinal features of neuroinflammation associated with acute and chronic neurological disorders (47, 48). As such, the efficacy of the STEP-mimetic to attenuate PGE_2_ release establishes the role of STEP as a regulator of neuroinflammatory response under excitotoxic conditions. This interpretation is further supported by recent findings in a STEP KO mouse model of ischemic stroke demonstrating that in the absence of STEP, increased microglial activation, blood–brain barrier disruption, and extravasation of immunoglobulins in the brain lead to exacerbation of ischemic brain injury (49). The study further showed that restoration of the STEP signaling with post-stroke administration of the STEP-mimetic resulted in attenuation of microglial activation and BBB disruption and significant reduction in infarct size in the STEP KO mice. Collectively, these findings highlight the role of STEP in neuroimmune communication and could lead to a paradigm shift in our understanding of neuroinflammatory disorders related to excitotoxicity.

The study further implies that the loss of function of endogenous STEP with aging could be a contributing factor for the enhanced inflammatory response characteristic of neurodegenerative diseases associated with aging. Because aging is associated with significant reduction in cerebral blood flow (50, 51, 52), the resulting hypoxia could increase the susceptibility of the aged brain to oxidative stress (53, 54). Such prooxidative shift, primarily due to the depletion of the brain glutathione level, has been shown to increase the dimerization and subsequent loss of function of endogenous STEP in aged rats (19). Additional studies in neuronal cultures have also confirmed that depletion of cellular glutathione level using diethyl maleate leads to dimerization and subsequent loss of function of STEP in neurons (19, 55). As STEP is a neuroprotectant, such loss of function of endogenous STEP combined with an increase in extracellular glutamate level during brain pathologies could exacerbate disease progression by enhancing inflammatory response in the aging brain. In conclusion, the study establishes the novel role of a tyrosine phosphatase in regulating neuronal inflammatory response. The findings also implicate the STEP-derived peptide mimetic as a promising new tool for targeting neuroinflammation in CNS pathophysiology.

## Experimental procedures

### Materials and reagents

Colonies of STEP KO mice, which were developed on a C57BL6 background, and the WT control mice (C57BL6) are maintained at the animal care facility of the University of New Mexico and were used to generate both WT and STEP KO timed pregnant mice (11). All procedures involving animals follow the ARRIVE guidelines and were approved by the Institutional Animal Care and Use Committee of the University of New Mexico Health Sciences Center. For tissue culture, glutamate, NMDA, glycine, and cytosine D-arabinofuranoside were purchased from Sigma-Aldrich. All other tissue culture reagents were obtained from Invitrogen. The bicinchoninic acid protein estimation kit and SuperSignal West Pico chemiluminescent reagent were purchased from Thermo Fisher. Selective pharmacological inhibitors were obtained as follows: MK-801 hydrogen maleate (MK801) from Tocris; SB 203580 and Bengamide B from EMD Biosciences; and CAY10404 from Cayman Chemical. Antibodies used for the study were as follows: rabbit monoclonal anti-phospho-p38 (T^P^EY^P^; Cat #: 9215), polyclonal anti-p38 (Cat #: 9218), and monoclonal anti-IκBα (Cat #: 4814) antibodies from Cell Signaling Technology; polyclonal anti-COX-2 antibody (Cat #: ab15191) from Abcam; monoclonal anti-STEP antibody (Cat #: NB300-202) from Novus Biologicals; and monoclonal anti-β-tubulin antibody (Cat #: T0198) from Sigma-Aldrich. Horseradish peroxidase–conjugated goat anti-rabbit (Cat #: 7074) and goat anti-mouse (Cat #: 7076) secondary antibodies were obtained from Cell Signaling Technology. The PGE_2_ ELISA kit was purchased from Arbor Assays (Cat #: K051-H1), and the cPLA2 activity assay kit was obtained from Cayman Chemical (Cat #: 765021).

### Purification of TAT-STEP-myc peptide

A recombinant DNA construct for TAT-STEP-Myc peptide was generated using a bacterial expression vector, expressed in *Escherichia coli* and purified as described previously (23, 49). Briefly, the nucleotide sequence of STEP_61_ cDNA encoding 173 to 279 amino acid was subcloned into a pTrc-His-Myc-TOPO expression vector (Invitrogen). A 11-amino acid TAT peptide (trans-activator of transcription of human immunodeficiency virus) nucleotide sequence was inserted at the N terminus of the STEP-Myc cDNA to render the peptide cell permeable. A point mutation was introduced by site-directed mutagenesis (Pfu Turbo, Stratagene) at serine 221 within the KIM domain (S221A) to render the peptide constitutively active in terms of its ability to bind to its substrate (13, 23, 43, 45). Two additional point mutations were also introduced at threonine 231 (T231E) and serine 244 (S244E) in the kinase specificity sequence domain to mimic the phosphorylated form that helps maintain the stability of STEP (43). The modified TAT-STEP-Myc peptide was expressed in *E. coli* and purified using BD Talon resin (BD Biosciences).

### Cell culture and stimulation

Embryos from WT and STEP KO mice (15- to 16-day gestation) were used to establish primary neuronal cultures, as described previously (45). Briefly, the cortex from the embryonic brain was dissected and the tissue dissociated mechanically and resuspended in Dulbecco's modified Eagle's medium/F-12 (1:1) containing fetal calf serum. Cells (6 × 10^6^ cells/dish) were plated on poly-D-lysine–coated 60-mm culture dishes and grown for 12 to 14 days in a humidified incubator (95:5% of air/CO_2_ mixture) at 37 °C. Three days after plating, cultures were treated with cytosine D-arabinofuranoside (10 μM) for 24 h to inhibit proliferation of non-neuronal cells. For neuronal stimulation, cells were washed twice with minimal essential medium followed by treatment with glutamate (50 μM) or NMDA (50 μM) for the indicated times. For some experiments, cells were returned to their original medium after treatment with glutamate for recovery. In some cultures, a selective pharmacological inhibitor MK801, SB 203580, CAY10404, or Bengamide B was added 15 min before treatment with glutamate and maintained throughout the duration of the experiment. In a subset of experiments, TAT-STEP-myc peptide (4 μM) was added 30 min before glutamate treatment. Cells were harvested at the specified time after stimulation and processed for immunoblot analysis or cPLA2 activity assay. The culture media were processed for measurement of PGE_2_ levels.

### Immunoblotting

For immunoblotting studies, neuronal cultures were washed with PBS (pH 7.4) containing sodium pyrophosphate and sodium vanadate as phosphatase inhibitors, and then harvested in SDS sample buffer (56), boiled at 100 °C for 10 min and centrifuged at 14,000*g* (10 min). An equal amount of protein (estimated using bicinchoninic acid kit) from the supernatant was processed for SDS-PAGE and immunoblotting. All primary antibodies and horseradish peroxidase–conjugated secondary antibodies were used according to manufacturer’s recommendations. Immune complexes were detected on X-ray film after treatment with the SuperSignal West Pico chemiluminescent reagent, and densitometric analysis of phosphorylated p38 MAPK, COX-2, and IκB was performed using the NIH ImageJ software.

### Measurement of cPLA2 activity

For measurement of cPLA2 activity, cells were washed and harvested in ice-cold Tris-buffered saline (pH – 7.4) containing phosphatase inhibitors. They were then sonicated (5 s bursts, three times with 2 min on ice between each burst), and the lysates centrifuged at 10,000 rpm (10 min) to obtain the supernatant. Equal amount of protein from the supernatants were processed for cPLA2 activity assay according to the manufacturer’s protocol. The assay is based on hydrolysis of the synthetic substrate, Arachidonoyl thio-PC by PLA2, leading to the release of a free thiol, which is detected by 5,5′-dithio-bis(2-nitrobenzoic acid) (DTNB). Briefly, the assay was performed in a microtiter plate by adding a fixed amount of the sample (or standard PLA2) and assay buffer in each well. The reaction was initiated by adding the substrate (Arachidonoyl Thio-PC) solution. After 60 min of incubation at room temperature (RT), the enzyme catalysis was stopped by adding DTNB/EGTA (5 min incubation at RT). The absorbance was measured at 414 nm using a plate reader.

### Measurement of PGE_2_ levels

To measure the PGE_2_ level released from neurons, culture mediums were collected from each experimental plate and centrifuged at 1000 rpm for 5 min to remove cellular debris. An equal volume (100 μl) of the supernatant from each sample was used for PGE_2_ assay using the PGE_2_ ELISA kit from Arbor assays and according to the manufacturer’s protocol. Briefly, the assay was performed in a microtiter plate coated with goat-anti-mouse IgG to capture a mouse mAb-specific for PGE_2_. The reaction was initiated after addition of a fixed amount of sample (or standard PGE_2_), HRP-conjugated PGE_2_, and the antibody against PGE_2_ to the plate. The assay is based on a competitive binding technique in which PGE_2_ present in the sample competes with the HRP-conjugated PGE_2_ for sites on the bound mouse mAb. After a 2 h incubation with shaking at RT and a brief washing to remove unbound sample and excess HRP-conjugated PGE_2_, a substrate solution was added to the wells to determine the enzyme activity of the bound HRP-conjugated PGE_2_. The intensity of the color was measured at 450-nm wavelength and is inversely proportional to the concentration of PGE_2_ in the sample.

### Statistical analysis

Statistical analysis was conducted with GraphPad Prism 9.0 software. All quantitative data are expressed as the mean ± SD. Statistical differences between multiple groups were assessed using one-way ANOVA followed by Tukey’s post hoc comparisons test. Mean differences between two groups were considered statistically significant when *p* < 0.05.

## Data availability

All data are contained within the article.

## Conflict of interest

The authors declare that they have no conflicts of interest with the contents of this article.

## References

[1] Lewerenz J., Maher P. (2015). Chronic glutamate toxicity in neurodegenerative diseases-what is the evidence?. Front. Neurosci..

[2] Olney J.W. (1986). Inciting excitotoxic cytocide among central neurons. Adv. Exp. Med. Biol..

[3] Lodge D. (2009). The history of the pharmacology and cloning of ionotropic glutamate receptors and the development of idiosyncratic nomenclature. Neuropharmacology.

[4] Spooren W., Lesage A., Lavreysen H., Gasparini F., Steckler T. (2010). Metabotropic glutamate receptors: Their therapeutic potential in anxiety. Curr. Top. Behav. Neurosci..

[5] Waxman E.A., Lynch D.R. (2005). N-methyl-D-aspartate receptor subtype mediated bidirectional control of p38 mitogen-activated protein kinase. J. Biol. Chem..

[6] Lynch D.R., Guttmann R.P. (2002). Excitotoxicity: Perspectives based on N-methyl-D-aspartate receptor subtypes. J. Pharmacol. Exp. Ther..

[7] Cuenda A., Rousseau S. (2007). p38 MAP-kinases pathway regulation, function and role in human diseases. Biochim. Biophys. Acta.

[8] Ji R.R., Suter M.R. (2007). p38 MAPK, microglial signaling, and neuropathic pain. Mol. Pain.

[9] Krementsov D.N., Thornton T.M., Teuscher C., Rincon M. (2013). The emerging role of p38 mitogen-activated protein kinase in multiple sclerosis and its models. Mol. Cell. Biol..

[10] He J., Zhong W., Zhang M., Zhang R., Hu W. (2018). P38 mitogen-activated protein kinase and Parkinson's disease. Transl. Neurosci..

[11] Deb I., Manhas N., Poddar R., Rajagopal S., Allan A.M., Lombroso P.J., Rosenberg G.A., Candelario-Jalil E., Paul S. (2013). Neuroprotective role of a brain-enriched tyrosine phosphatase, STEP, in focal cerebral ischemia. J. Neurosci..

[12] Segura Torres J.E., Chaparro-Huerta V., Rivera Cervantres M.C., Montes-Gonzalez R., Flores Soto M.E., Beas-Zarate C. (2006). Neuronal cell death due to glutamate excitotocity is mediated by p38 activation in the rat cerebral cortex. Neurosci. Lett..

[13] Poddar R., Deb I., Mukherjee S., Paul S. (2010). NR2B-NMDA receptor mediated modulation of the tyrosine phosphatase STEP regulates glutamate induced neuronal cell death. J. Neurochem..

[14] Legos J.J., McLaughlin B., Skaper S.D., Strijbos P.J., Parsons A.A., Aizenman E., Herin G.A., Barone F.C., Erhardt J.A. (2002). The selective p38 inhibitor SB-239063 protects primary neurons from mild to moderate excitotoxic injury. Eur. J. Pharmacol..

[15] Lombroso P.J., Naegele J.R., Sharma E., Lerner M. (1993). A protein tyrosine phosphatase expressed within dopaminoceptive neurons of the basal ganglia and related structures. J. Neurosci..

[16] Boulanger L.M., Lombroso P.J., Raghunathan A., During M.J., Wahle P., Naegele J.R. (1995). Cellular and molecular characterization of a brain-enriched protein tyrosine phosphatase. J. Neurosci..

[17] Bult A., Zhao F., Dirkx R., Sharma E., Lukacsi E., Solimena M., Naegele J.R., Lombroso P.J. (1996). STEP61: A member of a family of brain-enriched PTPs is localized to the endoplasmic reticulum. J. Neurosci..

[18] Nguyen T.H., Paul S., Xu Y., Gurd J.W., Lombroso P.J. (1999). Calcium-dependent cleavage of striatal enriched tyrosine phosphatase (STEP). J. Neurochem..

[19] Rajagopal S., Deb I., Poddar R., Paul S. (2016). Aging is associated with dimerization and inactivation of the brain-enriched tyrosine phosphatase STEP. Neurobiol. Aging.

[20] Dong P., Zhao J., Zhang Y., Dong J., Zhang L., Li D., Li L., Zhang X., Yang B., Lei W. (2014). Aging causes exacerbated ischemic brain injury and failure of sevoflurane post-conditioning: Role of B-cell lymphoma-2. Neuroscience.

[21] Mattson M.P., Magnus T. (2006). Ageing and neuronal vulnerability. Nat. Rev..

[22] Poddar R., Rajagopal S., Winter L., Allan A.M., Paul S. (2019). A peptide mimetic of tyrosine phosphatase STEP as a potential therapeutic agent for treatment of cerebral ischemic stroke. J. Cereb. Blood Flow Metab..

[23] Paul S., Nairn A.C., Wang P., Lombroso P.J. (2003). NMDA-mediated activation of the tyrosine phosphatase STEP regulates the duration of ERK signaling. Nat. Neurosci..

[24] Minghetti L. (2004). Cyclooxygenase-2 (COX-2) in inflammatory and degenerative brain diseases. J. Neuropathol. Exp. Neurol..

[25] Strauss K.I., Marini A.M. (2002). Cyclooxygenase-2 inhibition protects cultured cerebellar granule neurons from glutamate-mediated cell death. J. Neurotrauma.

[26] Shen Y., Kishimoto K., Linden D.J., Sapirstein A. (2007). Cytosolic phospholipase A(2) alpha mediates electrophysiologic responses of hippocampal pyramidal neurons to neurotoxic NMDA treatment. Proc. Natl. Acad. Sci. U. S. A..

[27] Shelat P.B., Chalimoniuk M., Wang J.H., Strosznajder J.B., Lee J.C., Sun A.Y., Simonyi A., Sun G.Y. (2008). Amyloid beta peptide and NMDA induce ROS from NADPH oxidase and AA release from cytosolic phospholipase A2 in cortical neurons. J. Neurochem..

[28] Yamamoto K., Arakawa T., Ueda N., Yamamoto S. (1995). Transcriptional roles of nuclear factor kappa B and nuclear factor-interleukin-6 in the tumor necrosis factor alpha-dependent induction of cyclooxygenase-2 in MC3T3-E1 cells. J. Biol. Chem..

[29] Shi G., Li D., Fu J., Sun Y., Li Y., Qu R., Jin X., Li D. (2015). Upregulation of cyclooxygenase-2 is associated with activation of the alternative nuclear factor kappa B signaling pathway in colonic adenocarcinoma. Am. J. Transl. Res..

[30] Kaltschmidt B., Linker R.A., Deng J., Kaltschmidt C. (2002). Cyclooxygenase-2 is a neuronal target gene of NF-kappaB. BMC Mol. Biol..

[31] Guo R.M., Xu W.M., Lin J.C., Mo L.Q., Hua X.X., Chen P.X., Wu K., Zheng D.D., Feng J.Q. (2013). Activation of the p38 MAPK/NF-kappaB pathway contributes to doxorubicin-induced inflammation and cytotoxicity in H9c2 cardiac cells. Mol. Med. Rep..

[32] Ackerman W.E.t., Summerfield T.L., Vandre D.D., Robinson J.M., Kniss D.A. (2008). Nuclear factor-kappa B regulates inducible prostaglandin E synthase expression in human amnion mesenchymal cells. Biol. Reprod..

[33] Kaltschmidt B., Widera D., Kaltschmidt C. (2005). Signaling via NF-kappaB in the nervous system. Biochim. Biophys. Acta.

[34] Karin M., Ben-Neriah Y. (2000). Phosphorylation meets ubiquitination: The control of NF-[kappa]B activity. Annu. Rev. Immunol..

[35] Rajagopal S., Fitzgerald A.A., Deep S.N., Paul S., Poddar R. (2019). Role of GluN2A NMDA receptor in homocysteine-induced prostaglandin E2 release from neurons. J. Neurochem..

[36] Munoz L., Ammit A.J. (2010). Targeting p38 MAPK pathway for the treatment of Alzheimer's disease. Neuropharmacology.

[37] Kim E.K., Choi E.J. (2015). Compromised MAPK signaling in human diseases: An update. Arch. Toxicol..

[38] Kramer R.M., Roberts E.F., Um S.L., Borsch-Haubold A.G., Watson S.P., Fisher M.J., Jakubowski J.A. (1996). p38 Mitogen-activated protein kinase phosphorylates cytosolic phospholipase A2 (cPLA2) in thrombin-stimulated platelets. Evidence that proline-directed phosphorylation is not required for mobilization of arachidonic acid by cPLA2. J. Biol. Chem..

[39] Lin L.L., Wartmann M., Lin A.Y., Knopf J.L., Seth A., Davis R.J. (1993). cPLA2 is phosphorylated and activated by MAP kinase. Cell.

[40] Lasa M., Mahtani K.R., Finch A., Brewer G., Saklatvala J., Clark A.R. (2000). Regulation of cyclooxygenase 2 mRNA stability by the mitogen-activated protein kinase p38 signaling cascade. Mol. Cell. Biol..

[41] Svensson C.I., Hua X.Y., Protter A.A., Powell H.C., Yaksh T.L. (2003). Spinal p38 MAP kinase is necessary for NMDA-induced spinal PGE(2) release and thermal hyperalgesia. Neuroreport.

[42] Paul S., Snyder G.L., Yokakura H., Picciotto M.R., Nairn A.C., Lombroso P.J. (2000). The Dopamine/D1 receptor mediates the phosphorylation and inactivation of the protein tyrosine phosphatase STEP via a PKA-dependent pathway. J. Neurosci..

[43] Mukherjee S., Poddar R., Deb I., Paul S. (2011). Dephosphorylation of specific sites in the kinase-specificity sequence domain leads to ubiquitin-mediated degradation of the tyrosine phosphatase STEP. Biochem. J..

[44] Munoz J.J., Tarrega C., Blanco-Aparicio C., Pulido R. (2003). Differential interaction of the tyrosine phosphatases PTP-SL, STEP and HePTP with the mitogen-activated protein kinases ERK1/2 and p38alpha is determined by a kinase specificity sequence and influenced by reducing agents. Biochem. J..

[45] Poddar R., Rajagopal S., Shuttleworth C.W., Paul S. (2016). Zn2+-dependent activation of the Trk signaling pathway induces phosphorylation of the brain-enriched tyrosine phosphatase STEP: MOLECULAR BASIS FOR Zn2+-INDUCED ERK MAPK ACTIVATION. J. Biol. Chem..

[46] Zuniga A., Torres J., Ubeda J., Pulido R. (1999). Interaction of mitogen-activated protein kinases with the kinase interaction motif of the tyrosine phosphatase PTP-SL provides substrate specificity and retains ERK2 in the cytoplasm. J. Biol. Chem..

[47] Prinz M., Priller J. (2017). The role of peripheral immune cells in the CNS in steady state and disease. Nat. Neurosci..

[48] Graeber M.B., Li W., Rodriguez M.L. (2011). Role of microglia in CNS inflammation. FEBS Lett..

[49] Rajagopal S., Yang C., DeMars K.M., Poddar R., Candelario-Jalil E., Paul S. (2021). Regulation of post-ischemic inflammatory response: A novel function of the neuronal tyrosine phosphatase step. Brain Behav. Immun..

[50] Ainslie P.N., Cotter J.D., George K.P., Lucas S., Murrell C., Shave R., Thomas K.N., Williams M.J., Atkinson G. (2008). Elevation in cerebral blood flow velocity with aerobic fitness throughout healthy human ageing. J. Physiol..

[51] Candelario-Jalil E., Paul S. (2021). Impact of aging and comorbidities on ischemic stroke outcomes in preclinical animal models: A translational perspective. Exp. Neurol..

[52] De Vis J.B., Hendrikse J., Bhogal A., Adams A., Kappelle L.J., Petersen E.T. (2015). Age-related changes in brain hemodynamics: A calibrated MRI study. Hum. Brain Mapp..

[53] Macri M.A., D'Alessandro N., Di Giulio C., Di Iorio P., Di Luzio S., Giuliani P., Esposito E., Pokorski M. (2010). Region-specific effects on brain metabolites of hypoxia and hyperoxia overlaid on cerebral ischemia in young and old rats: A quantitative proton magnetic resonance spectroscopy study. J. Biomed. Sci..

[54] Ostergaard L., Engedal T.S., Moreton F., Hansen M.B., Wardlaw J.M., Dalkara T., Markus H.S., Muir K.W. (2016). Cerebral small vessel disease: Capillary pathways to stroke and cognitive decline. J. Cereb. Blood Flow Metab..

[55] Deb I., Poddar R., Paul S. (2011). Oxidative stress-induced oligomerization inhibits the activity of the non-receptor tyrosine phosphatase STEP61. J. Neurochem..

[56] Laemmli U.K. (1970). Cleavage of structural proteins during the assembly of the head of bacteriophage T4. Nature.

